# The Role of Fruit Surface Bloom in Consumer Preference for Blueberries: Sensory Evaluation and Multisensory Interactions

**DOI:** 10.3390/foods14030455

**Published:** 2025-01-30

**Authors:** Claudia M. Asensio, Mary Lu Arpaia, David Obenland

**Affiliations:** 1 Department of Food Science and Human Nutrition, University of Illinois Urbana-Champaign, 905 S Godwin Avenue, Urbana, IL 61801, USA; mlarpaia@ucanr.edu; 2 Department of Botany and Plant Sciences, University of California, Riverside, CA 92521, USA; 3 USDA, Agricultural Research Service, San Joaquin Valley Agricultural Sciences Center, 9611 South Riverbend Avenue, Parlier, CA 93648, USA; david.obenland@usda.gov

**Keywords:** fruit surface bloom, consumer behavior, flavor acceptance, vision, taste, blueberry varieties, blueberry quality

## Abstract

Three blueberry cultivars (‘San Joaquin’, ‘Jewel’, and ‘Snowchaser’) were harvested at commercial maturity and subjected to different degrees of fruit surface bloom removal (full natural bloom (FB), partial bloom (PB), and removed bloom (RB)) to assess the importance of the bloom to consumer preference. Sensory evaluation assessed flavor, appearance, and overall acceptance under different conditions (darkness and light). Panelists preferred berries with at least some degree of bloom, as influenced by appearance for all varieties, and in ‘Snowchaser’ and ‘Jewel’, flavor acceptance declined when the evaluation allowed the fruit to be visualized. Panelists were prompted to select the attribute most influencing their affective evaluations to better understand the basis for the differences due to the presence of bloom. FB and PB were rated as more appealing, except for ‘Jewel’, where PB was deemed unattractive. At the same time, bloom presence influenced firmness choices, and sweetness and tartness preferences were affected by both variety and evaluation conditions, suggesting complex interactions in multisensory perception. Principal component analysis reinforced the idea that treatments with bloom removal were less accepted overall, while ‘Snowchaser’ samples were consistently well-liked relative to the other two varieties. These findings highlight the importance of bloom presence in blueberry quality perception and consumer behavior.

## 1. Introduction

Blueberries are the second most popular berry in value and volume in U.S. production. U.S. production increased 283 percent between 2000 and 2002 and between 2018 and 2020. The value of production increased over the last two decades to USD 713 million in 2020. Due to the high prices in the fresh market, global production has grown [1]. The demand for blueberries has grown as more consumers are leading their lifestyles and leaning toward blueberries for their health benefits. The average per capita availability for 2018–2020 was estimated at 2.2 pounds per person, more than 500 percent higher than the average during 2000–2002. The domestic demand for blueberries has grown rapidly because of the establishment of USHBC (U.S. Highbush Blueberry Council), which promotes the health benefits of blueberry consumption and invests in research [1].

The light blue coloration of fresh blueberries results from the presence of a waxy white surface layer known as the bloom that overlays and modifies a nearly black cuticle underneath [2,3]. This waxy surface layer is present in several other fruits, including plums and grapes [4], and it is believed to be crucial in maintaining the quality of fruits. The structure of the bloom in blueberries is in the form of tubules or rodlets, which increase in amount during fruit initiation and maturation, eventually covering the fruit surface [5,6,7]. Contrasting results have been obtained regarding whether the bloom increases or decreases during postharvest storage [8,9].

Moisture loss, commonly measured as weight loss, is closely related to firmness, an essential quality parameter of blueberries [10]. Weight loss has been shown to increase when the bloom on the fruit is removed [4,8,11], indicating that the bloom plays a role in maintaining blueberry firmness. Chu et al. [8] also found that bloom removal reduced antioxidant levels and related enzyme activities, as well as enhanced reactive oxygen species and lipid peroxidation, suggesting a more profound role of the bloom in blueberry metabolism. It was indicated that the grape epicuticular wax also directly repels pathogen growth or allows the berry surface to be more readily cleaned of pathogens by water flowing over the hydrophobic surface [12].

Many factors can affect the presence of bloom on the fruit. Mukhtar et al. [4] found that bloom can be removed during fruit harvesting, transporting, and packing, resulting in an unattractive appearance. This includes the processes of grading and sizing (Family Tree Farms, Kingsburg, CA, personal communication). Studies have also been performed to develop coatings to help maintain postharvest fruit quality, but few have described how the coatings affect the surface bloom of blueberries [11]. Duan et al. [13] found that both washing the fruit and applying a coating led to a substantial loss of bloom. Another researcher also noted a loss of lightness in blueberries treated with different combinations of sodium alginate and pectin [14] but did not directly comment on the loss of bloom. Following the application of some coatings, such as chitosan, the blueberries can have an almost black appearance due to the complete removal of bloom [11]. Research is also ongoing to determine the feasibility of washing, spraying, or dipping fruit to enhance food safety or to limit decay [15,16], which potentially could alter the bloom. Color helps consumers determine the freshness or ripeness of fresh produce [17]. Shifferstein et al. [18] observed in carrots that a lower saturation of the orange color was associated with lower attractiveness and freshness by panelists. In contrast, a higher orange color was evaluated as artificial, with red carrots being spicy, and purple and yellow carrots being thought of as less nutritious and artificial. Paaki et al.’s [19] findings revealed that color and color combinations of salad mixes significantly influenced visual appeal, with vibrant, high-contrast salads being more visually attractive than pale, low-contrast options, and concluded that using vibrant colors and stimulating color combinations can enhance the visual appeal of food, encouraging consumers to choose and eat more vegetables.

The impact of the surface bloom on consumer acceptance of blueberries is inadequately studied. Saftener et al. [20] found a weak negative correlation between the color measurement L* and sensory acceptance, potentially indicating that lighter blueberries (more bloom) are preferred. A marketing study [21] examined which blueberry traits were most important to consumers and found that the researchers’ characterizations attempting to describe the bloom received a relatively low ranking from the consumers compared to other characteristics. They also noted the difficulty in finding descriptors for the bloom. In another study, blue color was found to be of importance to 70% of the participants [22], which could indicate a preference for at least some degree of bloom, but preference for bloom was not an attribute directly investigated. Previous studies have demonstrated how bloom removal impacts the color parameters of the fruit, making the surface much darker and more intense blue in plums and blueberries [2,6], and others correlated this change with water losses and post-harvest shelf life [2,11,23]. However, no previous research confirms (or denies) how consumer acceptance is affected or not by the presence (or absence) of this waxy coat.

Retronasal smell and taste together make flavors [17,24]. It is unclear how much our other senses—like sight and hearing—contribute to our perception of flavor and the acceptance or rejection of food. They might be a big part of the flavor or change how we experience this attribute. In this study, to directly assess the importance of bloom to consumer acceptance, the bloom was carefully removed either partially or totally, and then the resulting blueberries were compared to those with intact bloom using sensory and quality analysis. This study investigated how the visual blueberry bloom attribute presence (or absence) influenced consumers’ preference, flavor perception, and overall acceptance of different blueberry varieties. The relationships between quality parameters, sensory variables, and attributes were explored. This study offers valuable insights to help blueberry breeders, producers, and marketers create and promote varieties with ideal bloom, boosting consumer appeal and supporting industry growth. It provides a significant understanding of how handling and processing methods, like bloom removal, influence consumer acceptance.

## 2. Materials and Methods

### 2.1. Blueberries

Blueberries were harvested from an experimental field at the USDA Agricultural Research Service San Joaquin Valley Agricultural Sciences Center (USDA-SJVASC) in Parlier, CA. Fruits from three different cultivars (‘San Joaquin’, ‘Jewel’, and ‘Snowchaser’) were picked at the appropriate maturity stage each morning before their evaluation on 12–14 May 2022, 24–26 May 2022, and 7–9 June 2022, respectively. Berries were manually harvested into 907 g plastic clamshells, selecting those with perfect bloom, and then carefully arranged on a single layer on a 48 cm × 28 cm × 10 cm translucent plastic container to avoid any bloom removal and ease subsequent berry selection.

### 2.2. Treatments

**Figure 1:**
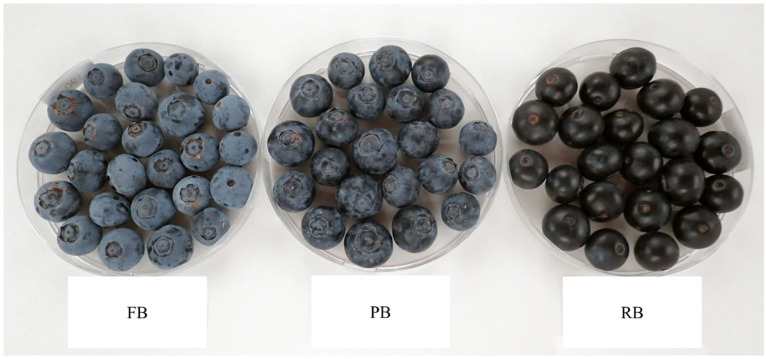
Examples of the appearance of blueberries with fully intact bloom (FB), partial bloom (PB), or blueberries where the bloom is totally removed (RB). Variety is ‘Snowchaser’.

To estimate the effect of bloom loss before their consumption, three different treatments using fruit without any apparent defects were prepared: (1) Full natural bloom (FB) refers to blueberries completely covered with bloom, without any visible dark spot of skin. (2) Partial bloom (PB) refers to the partially removed state attained by rolling a single layer of berries for 30 s over a 16-inch aluminum oven pizza pan to simulate handling. Several trials were performed to achieve a visible bloom removal that was an intermediate level between full bloom and total removal. (3) Removed bloom (RB) refers to the state in which the bloom was entirely manually removed from each treated berry with a Kimwipe (Kimberly-Clark, Rosewell, GA, USA) until the skin was fully visible, without any visible bloom (Figure 1). Great care was used to avoid damaging the fruit during this process. After berry sorting and treatment, the berries were held at room temperature (20 °C) for approximately 5 h when the panel was conducted. A new set of berries was harvested each day just prior to holding the sensory evaluation session.

### 2.3. Quality Analysis

Following each sensory panel, color and firmness were measured from the berries used for visual evaluation. Twenty berries were utilized for each parameter measurement. The color was determined using a Minolta CR-300 colorimeter (Ramsey, NJ, USA). Firmness was measured using a FirmTech2 (BioWorks, Wamego, KS, USA), and the force (in g) required to cause a 1 mm deflection to a berry was recorded.

### 2.4. Sensory Evaluation

#### 2.4.1. Panelists

Panelists were recruited through institutional emails from the University of California Kearney Agricultural and Extension Center (UC KARE) (Parlier, CA, USA) and USDA-SJVASC. A total of 60 (±1) people attended during three days of evaluations for each cultivar, with 20 sets of samples prepared daily.

People were allowed to participate following these criteria: (i) people without any food allergies, (ii) nonsmokers, and (iii) people above 18 years old. Internal Review Board Socio-Behavioral (IRB-SB) Exempt status for the panels was obtained from the UCR (IRB-SB HS-23-014).

The demographic composition of the panel is shown in Table 1. In general, the panel was balanced in terms of gender, and the ethnicity was representative of the Central California region. In terms of age, at least 65% of the people who attended the sessions were under 39. Regarding blueberry consumption, 35% of the people who participated in each session consume blueberries at least every two weeks.

#### 2.4.2. Test Design

Sensory evaluation sessions were conducted in the Sensory Evaluation Laboratory of UC KARE, equipped with six booths, a kitchen, and a conference room. Each sensory booth has a window with a sliding door connected to the kitchen where samples are prepared. The test instructions were explained to every panelist upon their arrival.

The experiment was fully designed, and data were collected using tablets connected to the cloud software Compusense^®^ Version 22.0.27 (Guelph, ON, Canada). Blueberries after bloom removal treatments were evaluated under two conditions: darkness and incandescent white light. In each condition, every treatment was tasted (FB, PB, and RB). Tasting samples consisted of 3 berries presented in 2 oz black plastic cups with lids (SOLO^®^ Cup Company, Urbana, IL, USA), labeled with three-digit number codes, and served at room temperature. Individuals were advised to eat the three berries in each sample at once. Each panelist tasted a total of 9 (3 × 3) 3-digit-coded samples. Visual samples were presented only in the light, consisting of a 10 cm Petri dish full of each treatment’s samples. The presentation order was randomized entirely within a condition. A bite of an unsalted cracker and a sip of water were used to clean their palates between samples (Supplementary File S1).

Panelists were informed that they were participating in a sensory acceptability test, and the instructions and conditions for the test were given. The objectives of the study and treatments were not unveiled to avoid any bias in their evaluations. First, panelists entered, and each sat at an individual booth, with serving trays already displayed on the counters covered with a paper towel. After that, the lights were turned off, creating a dark condition. Panelists could log in to their tablets and follow the test instructions. In darkness, panelists were asked to taste the samples and to focus only on flavor. Each sample was graded using a 9-point hedonic scale (from 1 (dislike extremely) to 9 (like extremely)). A select only one question was later displayed, asking them to choose one attribute (“the single most important”) that most influenced their decision from a list: (1) too soft, (2) firmness just right, (3) too hard, (4) too sweet, (5) sweetness just right, (6) not sweet at all, (7) too tart, (8) tart just right, (9) not tart at all.

After the panelists finished their evaluations, the lights were turned on, leading to the second condition: light (provided by an incandescent bulb). New trays consisting of two sets of samples, three visual samples, and three tasting samples were presented to each individual (all samples number coded). Panelists were advised not to touch or eat visual samples. Tasting samples were comprised of new paper cups with three more berries per sample to taste. In the first part of the test in the light, panelists were advised to focus on the first set of visual samples and grade their overall acceptance and color using a 9-point hedonic scale. After this, they were instructed to taste the second set of samples, focusing only on the taste, and grade it using a 9-point hedonic scale. Following the tasting, the question asking the “single most important” factor to be determined was presented, and it offered the following options: (1) appearances is appealing, (2) appearance is ok, (3) appearance is not attractive, (4) too soft, (5) firmness just right, (6) too hard, (7) too sweet, (8) sweetness just right, (9) not sweet at all, (10) too tart, (11) tart just right, and (12) not tart at all. The final part of this test involved an ordering question where panelists had to order the samples according to their purchasing intent.

### 2.5. Statistical Analysis

A total of 60 (±1) data points for each sample x treatment were collected. Results were expressed as mean ± standard deviations. Infostat and R Statistical software were used to analyze the data [25,26]. Analysis of variance (ANOVA, α = 0.05) and Fisher’s LSD multiple range tests were performed to determine significant differences between means. The effect of the variety (‘Snowchaser’, ‘Jewel’, and ‘San Joaquin’) and the treatment (FB, PB, and RB) on color, firmness, flavor, appearance, and overall acceptance were analyzed using mixed linear and general models (MLGMs), with variety and treatment being the fixed effects. The Friedman test was used to calculate mean ranks and establish differences among treatments for a variety (T^2^, α = 0.05) of purchasing-intention data. Relative-percentage data of attribute choices (appearance, firmness, sweetness, and tartness) were analyzed with MLGM to analyze the effects of the treatment and the condition in which samples were evaluated and to explore their interactions, means, standard deviations, and significant differences as calculated by (Fisher’s LSD, α = 0.05). MLGM was selected to analyze datasets with unequal numbers of observations, permit the evaluation of several effects across a complex experimental design that involved several sessions with different panelists, and allow the evaluation of the interaction of those effects within the experiment. The individual value of each treatment (FB, PB, and RB) under the condition where it was evaluated (light or dark) and the selected most important attribute (between sweetness, tartness, and firmness choices) were plotted on a heatmap. Cluster analysis was performed between bloom treatments (FB, PB, and RB) and evaluation conditions (light vs. dark) and plotted into a dendrogram. Principal component analysis (PCA) was performed on the correlation matrix of the standardized (normalized) data from the sensory and quality study [27]. PCA assessed associations between samples and sensory and quality variables. A biplot from PCA was obtained. Vectors and points represented dependent variables and treatments, respectively. The angle formed between vectors indicates a correlation between variables.

## 3. Results and Discussion

### 3.1. Quality Analysis

Color and firmness were measured daily using 20 berries per treatment from prior visual sensory testing. Significant color differences (measured as L-value) were observed among treatments for the studied varieties (Table 2). The color of blueberries is contingent upon their maturity level, and the presence of bloom can modify the visual perception of their color. It was previously observed that the fruit exhibited higher L* values and a lighter color on the day of harvest because of the presence of bloom [28]. Similar results were observed in this study, where blueberries with less bloom (PB and RB) were darker and had correspondingly lower L* values (Figure 1).

Blueberries with RB were less firm in all varieties, while PB was also less firm than FB in ‘San Joaquin’ (Table 2). Water loss is tied to firmness [10], but it seems unlikely that this was the reason for the differences. Obenland et al. [10] found that the weight loss of ‘Jewel’ blueberries with the bloom removed was 0.8% greater than blueberries with intact bloom over a period of 1 day at ambient conditions (20 °C). Given the much shorter amount of time (≈5 h) in this study that the blueberries remained in a state of altered bloom at a similar temperature, it is likely that the weight loss was much less and not a factor in the firmness differences seen, given that blueberry softening begins at ≥2% [10]. Even though great care was given in removing the bloom, it is most likely that the differences in firmness were due to the mechanical action (rolling or rubbing the berries) required to remove the bloom. Firmness measured instrumentally was related to the degree of bloom present on the blueberries, but it is not apparent that this impacted flavor. The liking of RB, PB, and FB was equivalent when the fruits were viewed in darkness, but under light, RB was significantly less liked, indicating that visual perception, rather than differences in firmness, were most responsible.

### 3.2. Sensory Analysis

#### 3.2.1. Flavor, Visual, and Overall Analysis

The flavor quality of blueberries is a characteristic governed by genetic factors and strongly influenced by the environment, as evidenced by prior studies [29]. This assertion is further supported by the substantial variations in flavor characteristics observed among numerous blueberry cultivars [10,29,30]. In this study, panelists first evaluated blueberry flavor under darkness and later in the presence of light (Table 3). The first condition was set up to assess the effect of the presence of bloom on the flavor with no visual input, and the second was to evaluate the impact on the overall appreciation of the fruit when the presence of bloom could be clearly observed. In the darkness, panelists did not detect significant differences in flavor among treatments (*p* > 0.05) (FB, PB, and RB) in ‘Snowchaser’ and ‘San Joaquin’ berries. However, significant differences in the flavor acceptability of ‘Jewel’ berries were observed, with RB having the highest acceptance (7.67) and being significantly different from PB but not the FB treatment. Different results were observed when panelists could evaluate the flavor of the berries in the presence of light. Differences were observed for ‘Snowchaser’, with PB and FB being the most liked, although the differences between FB and RB were not statistically significant. A similar result was observed in ‘Jewel’, where RB treatment had the lowest score, which, in this case, is statistically the same as that for PB. For ‘San Joaquin’, no differences were observed in the light. Many food companies sometimes overlook the fact that altering the color of their products for any reason can impact on how the food tastes to consumers. Several studies were set up to study the discrepancy between the flavor that an individual associates with a color and the actual flavor experienced. As an example, in a study, the blue color of a commercial white-potato soup negatively impacted willingness to eat, palatability, comfort, and warmth ratings, and it increased anxiety compared to white and yellow soups. After eating, blue soup resulted in lower satiety ratings and a tendency for lower thermal sensation scores compared to white and yellow soups [31,32,33]. The importance of appearance to flavor appears to be indicated for blueberries as well, in this case focusing on the impact of the bloom. This is particularly important since it has been highlighted that bloom can be removed during key post-harvest processes of blueberries, such as harvesting, transporting, grading, sizing, and packing, if the fruit is not handled with great care. Kteniodaki et al. [23] evaluated fruit appearance as a quality attribute that influences shelf life and waste, finding that it declined during storage across all treatments. Blueberries stored under control conditions had the highest scores, while those exposed to simulated supply-chain conditions, particularly extreme conditions, generally received lower appearance scores. The effect of the condition (light versus dark) was also analyzed within each condition in each variety. Significant differences (*p* < 0.05) were detected in RB ‘Snowchaser’ and ‘Jewel’ when the condition changed from dark to light, suggesting that panelists are subjective when evaluating the taste and being able to see the fruit (Table 3). Their ability to see the fruit influenced panelists’ taste perception, suggesting a subjective element tied to visual cues during sensory evaluation. These findings highlight the importance of visual appearance in shaping consumer perception and preferences, emphasizing the role of lighting conditions in food presentation. For the food industry, this underscores the potential to leverage visual appeal in product marketing and packaging to enhance consumer experiences, particularly for fresh produce and light-sensitive foods. This seems similar to how the attraction (or rejection) to uncommonly colored fruits and vegetables is mainly because they look different, not necessarily because they taste different. An example is white strawberries (also called pine berries) [34]. Panelists’ purchasing intention reflected these results (Table 3). The lowest mean value (of the ranks) means that that sample was chosen more times as the first option. For ‘Snowchaser’ and ‘Jewel’ berries, panelists would prefer to purchase samples with either FB or PB, as these two treatments significantly differ from RB. Conversely, RB, with the highest mean rank, is considered the worst-performing group in terms of purchase intent. Significant differences in purchase intent were noted for ‘Snowchaser’ and ‘Jewel’, with RB being less preferred. Although ‘San Joaquin’ blueberries demonstrated the same trend, no significant differences were present between the levels of bloom removal. A significant positive correlation (Pearson’s coefficient r = 0.84, *p* > 0.05) was found by combining all the experiment data between overall acceptance and appearance. Vision is suggested to play an indirectly dominant role in multisensory flavor perception. This is attributed to its function in establishing taste and flavor expectations, which in turn serve as an anchor for the subsequent taste experience [17]. As an example, consumers for a long time have relied on color cues to assess the freshness and ripeness of produce [33].

**Figure 2:**
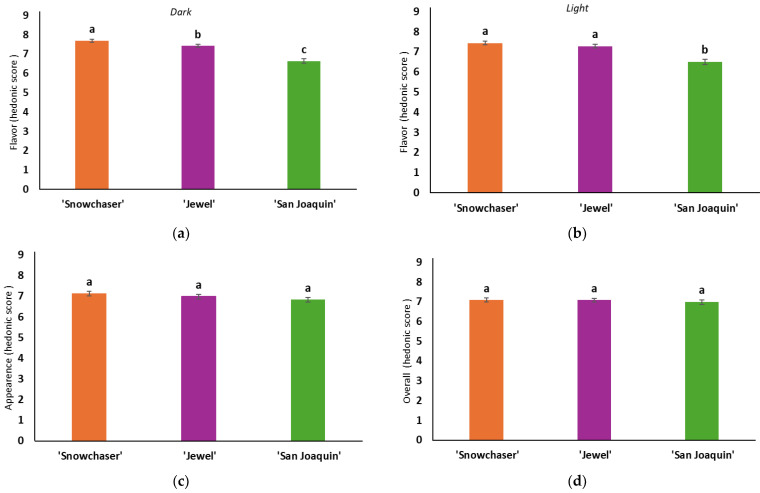
Varietal effect (‘Snowchaser’, ‘Jewel’, and ‘San Joaquin’) on the flavor (in dark (**a**) or light (**b**)), appearance (**c**), and overall acceptance (**d**). Different letters represent significant differences (*p* < 0.05) between varieties (Snowchaser, Jewel, and San Joaquin).

**Figure 3:**
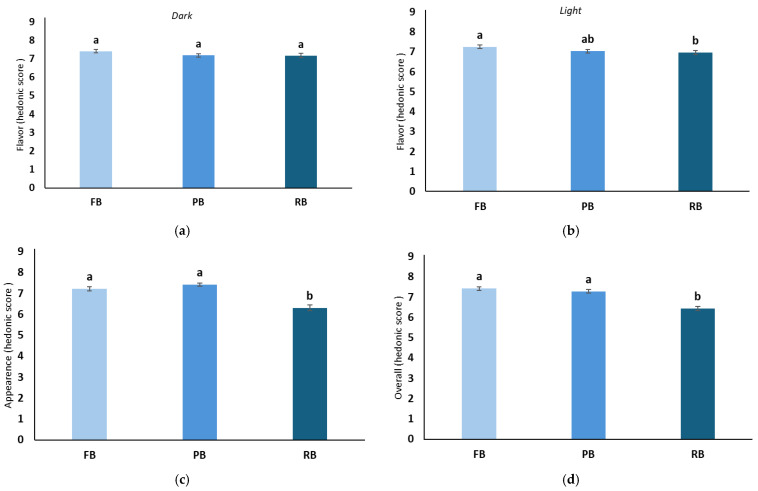
Effect of the bloom treatment (full bloom (FB), partial bloom (PB), and removed bloom (RB)) on the flavor (in dark (**a**) or light (**b**)), appearance (**c**), and overall acceptance (**d**). Different letters represent significant differences (*p* < 0.05) between treatments (FB, PB, and RB).

A mixed linear and general model (MLGM) was used to determine whether significant sources of variation were caused by each separate fixed effect (varieties and treatments) on the hedonic perception of flavor, appearance, and overall acceptance. The results of this analysis were used to plot Figure 2; Figure 3, respectively. When samples were analyzed in the dark, there were significant differences in the flavor among all varieties detected by panelists (*p* > 0.001) (Figure 2a). However, when the same samples were tasted under light, panelists were not able to differentiate between the flavors of ‘Snowchaser’ and ‘Jewel’, but they did distinguish these from ‘San Joaquin’ (Figure 2b). In a previous study, experts’ descriptions of the aroma of a red-colored, barrique-fermented young Chardonnay were more precise when served in an opaque glass compared to clear glass [35]. Both the color and intensity of color seem to automatically create expectations in the observer’s mind regarding the probable identity and intensity of the taste and flavor of food and drink. Regarding the effect of the treatments on the flavor, no significant differences were observed under dark conditions (Figure 3a). Still, the treatment affected the flavor perception in the light (Figure 3b). It has been observed that food characterized by flavors strongly associated with specific colors may be more susceptible to the influence of color cues compared to flavors with weaker color associations (e.g., salty-tasting products). Moreover, the taste and flavor perception in foods with intricate flavor profiles might be more influenced by visual cues than in foods with more straightforward taste profiles (e.g., water with added sugar) [36].

The panelists were asked to grade the visual appearance of the fruit. No significant effect of the variety was obtained (‘Snowchaser’, ‘Jewel’, and ‘San Joaquin’ varieties, (Figure 2c). However, samples with FB and PB were rated significantly higher than samples with RB, meaning that panelists least liked the RB treatment, and significant differences were found between the treatments (Figure 3c). This is following Motoki et al. [36], who stated that the intrinsic visual cues might carry information on the physical properties of foodstuffs, and they may be informative about the flavor. In this study, the association could be that a blueberry with at least some bloom is more flavorful than blueberries lacking bloom. This can be comparable to the peel color, where oranges with orange color sold better than green-color skin varieties in which flesh was equally flavorful, but the visual appearance of the fruit influenced food marketers and breeders [37]. In previous research, the presence of surface bloom (wax) influenced the L* value, potentially resulting in lighter fruit, and may have also played a role in shaping the visual perception of fruit color, particularly in blue chroma [20]. However, it is important to note that the study did not specifically assess the presence of surface bloom (wax) or directly assess its impact on acceptance. The presence/absence of bloom seemed to affect the overall perception of the berries (Figure 3d). These results followed the visual appearance results. The overall acceptance of samples with FB and PB was higher and significantly different from that of samples with RB for all varieties (Table 3). Similarly to the visual evaluation, the overall rating presented no significant differences due to the variety (Figure 2d). However, the effect of the bloom treatment did cause significant differences in the overall acceptability of the berries (Figure 3d). Most research suggests that changing the color of food or beverage can affect the perceived flavor, but not all studies agree. Altering color intensity can sometimes impact taste and flavor intensity, but only sometimes [17]. These differences in findings may be due to varying taste and flavor expectations associated with food colors among individuals. The effect of removing or not removing the bloom seems essential not only in consumers’ affective acceptance of the fruit but also has been demonstrated to be crucial in maintaining blueberries’ post-harvest shelf life. A Pearson’s correlation analysis revealed that to attain a high-quality postharvest flavor in blueberries, it is essential to preserve the fruit’s firmness throughout the storage period [38].

#### 3.2.2. Effects of Bloom Removal on Fruit Quality Perception

The three critical attributes of blueberry fruit quality—sweetness, tartness, and firmness—can be accurately assessed using basic laboratory equipment or human perception [39]. Panelists were forced to choose an attribute that most influenced their affective evaluations (Table 4). Choices were first grouped into three major categories: appearance (included (1) appearances is appealing, (2) appearance is ok, and (3) appearance is not attractive); firmness (included (1) too soft, (2) firmness just right, and (3) too hard); sweetness (included (1) too sweet, (2) sweetness just right, and (3) not sweet at all); and tartness (included (1) too tart, (2) tart just right, and (3) not tart at all). For samples evaluated under light, ‘Snowchaser’ registered 39.17% of the votes for appearance. This percentage is significantly different in this category from the other varieties. Meanwhile, for ‘Jewel’ and ‘San Joaquin’ berries, flavor attributes (sweetness, 51.7%; and tartness, 41.67%, respectively) were picked as the most important attributes that condition affective perception. Similar results were observed in darkness (where appearance was not evaluated). Appearance choice was significantly affected (*p* < 0.05) by the variety of fruit (Table 4), explaining the variability among data. People who chose firmness as the main trait were influenced by the light condition where the berries were evaluated (*p* < 0.0002). Regarding sweetness and tartness, both choices were influenced by variety, but light condition only influenced the sweetness choice. These results suggested that when panelists were allowed to see the blueberries, firmness and sweetness choices were affected, probably because more attention was given to appearance.

**Figure 4:**
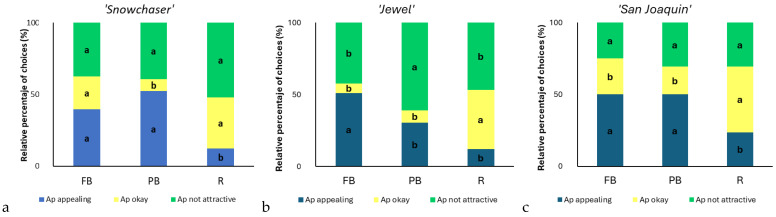
Distribution of appearance (Ap) choices relative percentages for ‘Snowchaser’ (**a**), ‘Jewel’ (**b**), and ‘San Joaquin’ (**c**) blueberry samples with different bloom content (FB, full bloom; PB, partial bloom; and RB, removed bloom) evaluated in the presence of light. Different letters represent significant differences (*p* < 0.05) between treatments within an appearance choice (appearance appealing, appearance okay, and appearance is not attractive).

In multisensory flavor perception, vision seems to play an indirectly dominant role by aiding in establishing taste and flavor expectations, which then serve as a foundation for the subsequent taste experience [17]. The distribution of the appearance choices was slightly different among varieties (Figure 4). In general, “appearance is appealing” received higher relative percentages in berries with FB and PB for all varieties except for ‘Jewel’, where “Ap not attractive” was predominant for PB. RB treatment, on the other hand, was most commonly characterized as “Ap okay” or “Ap not attractive” in all the varieties. Visual aspects such as color, shape, and visual texture have received special attention in the context of food products. It is important to note that many consumers associate bright colors with artificially manipulated food, while sometimes food products are given “special” colors to capture consumer’s attention [34].

**Figure 5:**
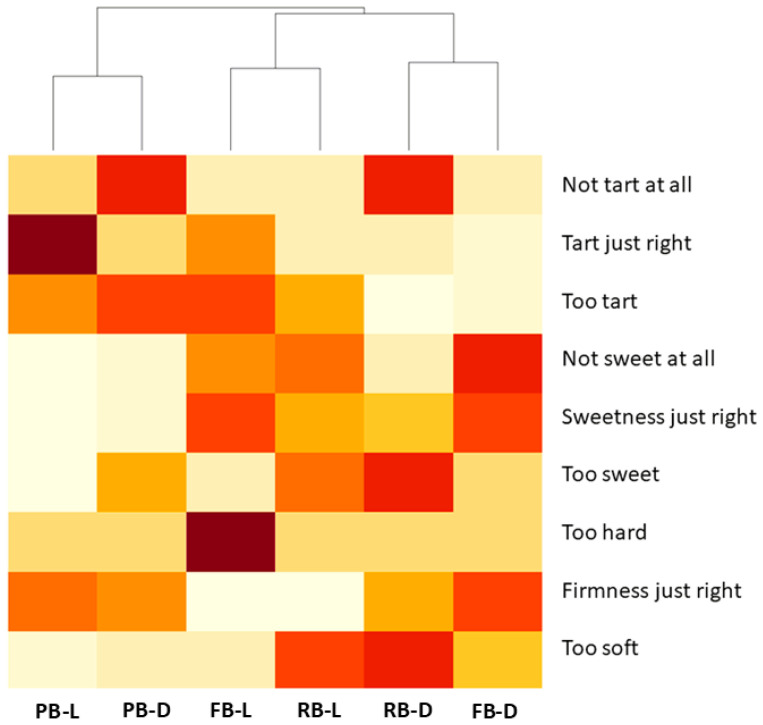
Heatmap matrix displaying the firmness, sweetness, and tartness choices across bloom treatments (FB, full bloom; PB, partial bloom; RB, removed bloom) combined with evaluation condition (L (light) vs. D (dark)), accompanied by a dendrogram grouping treatments based on similarity algorithm. Color power: Darker colors in the graph mean a specific trait was selected with a higher frequency for that treatment.

To determine the effect of the bloom treatments and the evaluation conditions on people’s choices, data were analyzed without the effect of variety, and treatments were combined with the evaluation conditions (FB light, PB light, RB light, FB dark, PB dark, and RB dark). The appearance was not included in the dataset, as it was only analyzed in the presence of light. Figure 5 shows the results of a heat map matrix of the firmness, sweetness, and tartness choices with treatments and a dendrogram where these were grouped by similarity.

The hierarchy clustering of the treatments showed that PB light grouped with PB dark (group 1), FB light and R light (group 2), and RB dark and FB dark (group 3), denoting that the intermediate bloom conditions lead to specific choices, whereas the presence or absence of light was determinant for others. In partial bloom treatments (group 1), participants focused their attention on firmness (just right) and tartness choices (the darker the color, the more votes the choice got). Surprisingly, panelists paid little or no attention to the sweetness in this bloom condition. In the presence of light (group 2), panelists focused their attention more on sweetness choices and less on tartness, and the number of votes (intensity of color) that each category got was, in general, moderate and depended on the bloom treatment. Interestingly, FB light received the highest number of votes for being “too hard”; in addition, a lower number of votes designated the treatment as being “too soft”. When FB and RB treatments were evaluated under darkness (group 3), firmness choices were predominant, indicating that panelists “focused” more on a physical attribute when samples were not visible. RB dark blueberries were the most voted as “too soft”. Interestingly, examining all treatments, we found that a higher number of votes were recorded for the extremes of the scale, either in sweetness (“too sweet” or “not sweet at all”) or tartness (“not tart at all”) choices with respect to the same bloom treatment but evaluated in light presence, meaning that panelists perceived those flavors in darkness as more intense. In a previous study where the contributions to the overall liking of blueberries were analyzed, sweetness intensity had a significant positive correlation (R^2^= 0.70), whereas sourness (R^2^= −0.55) negatively affected the overall liking [29]. It is worth noting that samples that were “voted” to be sweet were not selected to be “tart” at the same time. This is expected due to the mixture suppression of sweet and sour tastes 40. Sweetness had previously exhibited a stronger correlation with soluble solids/titratable acidity than with soluble acidity alone (0.62) [39]. This phenomenon is likely because the perception of sweetness is influenced more by the sugar-to-acid ratio than by the total sugar content, as supported by previous studies in the literature [29,40,41]

### 3.3. Principal Component Analysis

**Figure 6:**
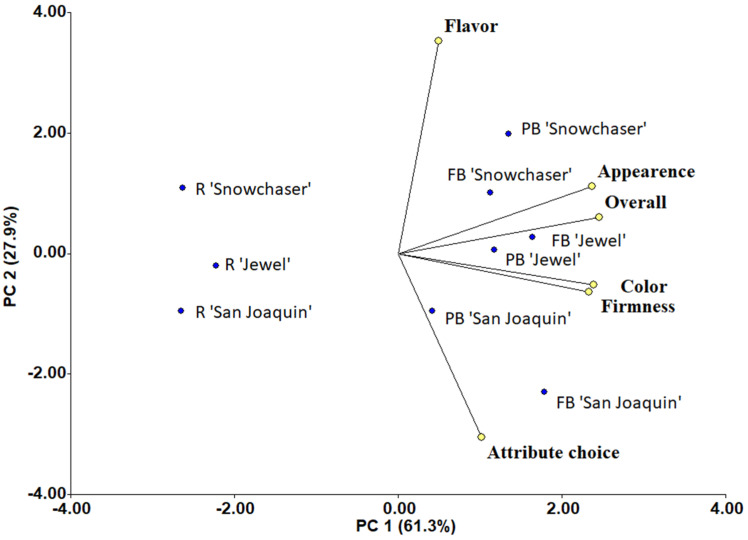
Biplot of the first (PC1) and second (PC2) components of the principal component analysis (PCA) of blueberry sensory evaluation in the presence of light. ‘Jewel’, ‘San Joaquin’, and ‘Snowchaser’ were the studied varieties, and FB (full bloom), PB (partial bloom), and R (removed bloom) were the treatments evaluated.

A principal components analysis (PCA) was performed using blueberry data from samples evaluated in the presence of light, and the biplot is presented in Figure 6. The two principal components of the PCA represented 89.2% of the variability of the data, with PC1 representing 61.3% of the variability. Vectors of dependent variables (flavor, appearance, overall acceptance, color (L-value), firmness, and attributes’ choice) had similar magnitude and were located on the plot’s right side. Similar orientations within some vectors (small angles between them) can be observed, showing a strong positive correlation. The overall acceptance of the berries was highly related to their appearance and flavor, as well as their color and firmness readings. Meanwhile, color and firmness readings were not associated with the flavor of a sample (vectors 90°), confirming what was previously observed: flavor is significantly influenced by the appearance of the berries. It depends on the varieties as well (Figure 2 and Figure 3).

The dispersion of the points indicated high variability among the data. Treatments with RB were placed on the left side of the plot, opposite to the other samples and the dependent variables, suggesting poor overall acceptance, appearance, flavor scores, and lower color and firmness readings. This agrees with the results from this study, where berries with removed bloom had lower firmness and color readings (Table 2). ‘San Joaquin’ (RB) was the furthest from the sensory variables (flavor, overall acceptance, and appearance), as it was the least liked variety by panelists (Figure 2c). ‘Snowchaser’ samples (PB, FB, and RB) were all placed on the upper side of the PC2, having high flavor ratings (Table 3). Samples with PB or FB were on the right side of the plot, grouped by variety rather than treatments. ‘Jewel’ samples (FB and PB) were associated with appearance, overall acceptance, color, and firmness vectors. In contrast, ‘San Joaquin’ samples (PB and FB) were placed on the lower right quadrant of the plot associated with choice, color, and firmness and far from the flavor variable due to a lower score. The choice of an attribute for these samples should be related to their firmness or color rather than to flavor attributes (sweetness/tartness). The results of this biplot are consistent with the results from the sensory analysis of this study, where appearance plays a vital role in panelist behavior, as well as flavor. The evaluated varieties had a unique flavor, but the quantity of bloom on the berries conditioned the panelists’ overall acceptance of them.

Although novel results were achieved in this study, it is essential to address its potential limitations. As a human sensory evaluation study, panelists may have varying preferences, familiarity with blueberries, or cultural biases that could influence their ratings. We acknowledge that our sensory panel, composed of individuals from our institution, may carry a potential risk of institutional bias. Although measures such as blind testing and standardized evaluation procedures were implemented to mitigate this, the potential for subconscious influence cannot be entirely dismissed. Our panel also included some members who were not frequent blueberry consumers, a factor which could impact on the generalizability of the findings, as their preferences and ratings may differ from those of habitual consumers. Nonetheless, the study offers valuable insights into the sensory attributes of blueberries and their appeal to a wider audience. The findings may not fully represent consumer preferences in diverse markets or geographic regions, as the panel composition might not reflect the broader population. How bloom and sensory descriptive attributes change over extended storage periods should be accounted for in the future, as such information could be critical for shelf-life considerations.

## 4. Conclusions

In this study, vision influenced panelists’ flavor and overall acceptance of blueberries, with berries exhibiting FB or PB being more favored by panelists than RB. This is the first study where blueberry bloom has been experimentally demonstrated to be involved in determining consumer preference. Blueberry breeders can incorporate the findings into their programs, seeking varieties with more bloom and good flavor profiles. Producers can find these results valuable, and marketers can use them to enhance consumer appeal and satisfaction, ultimately contributing to the promotion of blueberry consumption and industry growth. Consequently, increased blueberry consumption brings associated economic benefits. The information should also be valuable in evaluating the impact on consumer acceptability of processes that remove bloom from blueberries, such as handling and the application of coatings. The role of bloom should continue to be explored not only in blueberries but also in other fruits. In the future, studies examining how bloom influences storage, chemical parameters, and other sensory attributes should be performed to have a complex understanding of its importance.

## Figures and Tables

**Table 1:** The demographic composition (%) of each three-day sensory session.

	Session		
	‘Snowchaser’	‘Jewel’	‘San Joaquin’
**Gender**			
Female	50	60	50
Male	45	35	50
Prefer not to answer/other	5	5	
**Ethnicity**			
American Indian or Alaskan native	5		
Asian	35	35	30
Caucasian	5		15
East Indian			
Hispanic or Latino	30	45	40
Native Hawaiian or Pacific Islander			
Other/prefer not to answer	20	20	15
**Frequency of eating blueberries**			
Everyday		5	5
More than twice a week	5		10
Once a week	20	15	10
Every two weeks	10	15	10
Monthly	30	20	35
Less than monthly	30	40	30
I don’t eat blueberries	5	5	
**Age (years)**			
Under 19		5	
19–29	40	40	50
30–39	25	35	35
40–49	10	15	10
50–59	5	5	
60–69	20	15	5
70 or over			
Previous experience *			
Yes	90	90	100
No	10	10	0

* Panelists’ previous experience meant they had previously participated in sensory analysis tests.

**Table 2:** Evaluation of color (L-value) and firmness in blueberry varieties (‘Snowchaser’, ‘Jewel’, and ‘San Joaquin’) with full bloom (**FB**), partial bloom (**PB**), and removed bloom (**RB**).

	‘Snowchaser’ ^a^						‘Jewel’ ^a^						‘San Joaquin’ ^a^					
	FB ^d^		PB ^d^		RB ^d^		FB ^d^		PB ^d^		RB ^d^		FB ^d^		PB ^d^		RB ^d^	
**Color (L) ^b^**	41.19	**a**	35.04	**b**	25.93	**c**	42.37	a	35.02	**b**	26.71	**c**	45.31	**a**	37.01	**b**	26.54	**c**
**Firmness ^c^**	156.91	**a**	147.99	**a**	126.28	**b**	158.1	a	156.95	**a**	121.8	**b**	176.84	**a**	136.8	**b**	119.68	**b**

^a^ Different letters in a row show significant differences (*p* < 0.05) between means of each treatment per a variety. ^b^ Color: L-value data obtained with a colorimeter. ^c^ g/1 mm deflection. ^d^ Samples with FB (full bloom), partial bloom (PB), and removed bloom (RB).

**Table 3:** Means of flavor, overall acceptance, and color (visual) affective evaluations (on a hedonic scale of 1–9) of blueberry varieties (‘Snowchaser’, ‘Jewel’, and ‘San Joaquin’) with full bloom (**FB**), partial bloom (**PB**), and removed bloom (**RB**) under two evaluation conditions: light (**L**) and dark (**D**).

		‘Snowchaser’ ^a,b^						‘Jewel’ ^a,b^						‘San Joaquin’ ^a,b^					
		FB ^g^		PB ^g^		RB ^g^		FB ^g^		PB ^g^		RB ^g^		FB ^g^		PB ^g^		RB ^g^	
**Flavor ^c,d^**	D	7.65	**a 1**	7.63	**a 1**	7.78	**a 1**	7.38	**ab 1**	7.20	**b 1**	7.67	**a 1**	6.52	**a 1**	6.67	**a 1**	6.75	**a 1**
	L	7.43	**ab 1**	7.78	**a 1**	7.12	**b 2**	7.42	**a 1**	7.28	**ab 1**	7.15	**b 2**	6.23	**a 1**	6.68	**a 1**	6.62	**a 1**
**Overall ^c^**	L	7.45	**a**	7.62	**a**	6.45	**b**	7.4	**a**	7.37	**a**	6.47	**b**	7.27	**a**	7.27	**a**	6.40	**b**
**Color ^c^**	L	7.32	**a**	7.70	**a**	6.37	**b**	7.25	**a**	7.35	**a**	6.35	**b**	7.08	**a**	7.22	**a**	6.20	**b**
**Purchase intent ^e,f^**	L	1.83	**ab**	1.77	**a**	2.40	**c**	1.75	**a**	1.83	**ab**	2.42	**c**	1.87	**a**	1.95	**a**	2.18	**a**

^a^ Different letters in a row show significant differences (LSD-Fisher, *p* < 0.05) between means of each treatment per a variety. ^b^ Different numbers in a column show significant differences (LSD-Fisher, *p* < 0.05) between means of the same treatment under different conditions (D or L). ^c^ Sensory affective data, hedonic score. ^d^ Samples evaluated in darkness (D) or light (L). ^e^ Mean ranks of the Friedman test of purchasing intention data represent the average relative standing of each group across all the observations. A group with the lowest mean rank is the best-performing group, as it had the lowest average rank across all observations (was chosen more times as 1st option). ^f^ Mean ranks with a common letter indicate no significant differences (T^2^, *p* > 0.05). ^g^ Samples evaluated in darkness (D) or light (L).

**Table 4:** Results from “the single most important attribute” test (relative percentages %) grouped by appearance, firmness, sweetness, and tartness aspects evaluated under regular light (L) and darkness (D) and the factors influencing that choice.

		Appearance ^a,b^		Firmness ^a,b,c^		Sweetness ^a,b,c^		Tartness ^a,b,c^	
**‘Snowchaser’**	**L ^e^**	39.17	a	13.33	a	32.50	b	15.00	b
**‘Jewel’**	**L ^e^**	18.89	b	7.78	a	51.67	a	21.67	b
**‘San Joaquin’**	**L ^e^**	17.78	b	10.56	a	30.00	b	41.67	a
**‘Snowchaser’**	**D ^e^**	-		22.50	a	50.83	ab	26.67	a
**‘Jewel’**	**D ^e^**	-		16.11	a	62.22	a	21.67	a
**‘San Joaquin’**	**D ^e^**	-		22.78	a	43.89	b	33.33	a
**Darkness**		-		20.21	a	52.50	a	27.50	a
**Light**		-		10.21	b	38.75	b	27.29	a
***Factors ^d,e^***									
Variety		0.0002		ns		0.0003		0.0023	
Bloom removal (B)		ns		ns		ns		ns	
Light or dark (L)		na		0.0002		0.0012		ns	
Variety: B		ns		ns		ns		ns	
Variety: L		na		ns		ns		ns	
B:L		na		ns		ns		ns	
Variety: B: L		na		ns		ns		ns	

^a^ Appearance includes the choices appearance is appealing, appearance is okay, and appearance is not attractive; firmness consists of the choices too soft, firmness just right, and too hard; sweetness includes too sweet, sweet just right, and not sweet at all; tartness includes too tart, tart, and just right. ^b^ Different letters in a column indicate significant differences between varieties (α = 0.05, LSD-Fisher) for a given light condition. ^c^ Dependent factors and their interactions, *p*-value > 0.05 = not significant (ns). ^d^ na, do not apply; appearance was only evaluated in light presence. ^e^ Samples evaluated in light presence (L) or under darkness (d).

## Data Availability

The datasets generated from this research are available to anyone who requests them to the corresponding author.

## Supplementary Materials

The following supporting information can be download at https://www.mdpi.com/article/10.3390/foods14030455/s1, Supplementary File S1: A Compusense^®^ template of the test is included.
