# Supplementary material for: The Role of Fruit Surface Bloom in Consumer Preference for Blueberries: Sensory Evaluation and Multisensory Interactions
Source: Foods. 2025 Jan 30;14(3):455. doi: 10.3390/foods14030455 (PMC11817841; doi:10.3390/foods14030455)
Supplement: Supplementary file 1 [file foods-14-00455-s001.zip › foods-3390407-supplementary.pdf]

Welcome!

TODAY YOU WILL TASTE BLUEBERRIES!

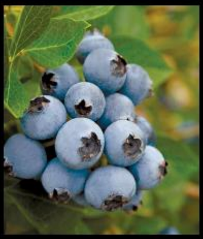

First, you will answer personal information questions.

Then you will do a two-section test:

1st section-test under darkness, followed by a 2nd section under regular light.

Please follow the instructions carefully, if you have any questions don't hesitate in asking for help!

After you click next, you will not be able to return to the previous screen.

Next

Please write your full name and email in the box below.

Please select your gender.

- Female
- Male
- Other
- Prefer not to answer

How do you identify yourself?

- American Indian or Alaskan native
- Asian
- Caucasian
- Easter Indian
- Hispanic or Latino
- Native Hawaiian or Pacific islander
- Other
- Prefer not to answer

How often do you eat blueberries?

- Everyday
- More than twice a week
- Once a week
- Every two weeks
- Monthly
- Less than monthly
- I don't eat blueberries

Have you participated in any sensory evaluation tests before?

- Yes
- NO

Please indicate your age.

- Under 19
- 19 - 29
- 30 - 39
- 40 - 49
- 50 - 59
- 60 - 69
- 70 or over

ACCEPTABILITY TEST: FLAVOR

SAMPLE BC111

Focus **only on the FLAVOR** of each sample.
Please **eat all the blueberries that are in the paper cups** at once and express how much you like or dislike them.

Dislike extremely	Dislike Very Much	Dislike Moderately	Dislike Slightly	Neither Like nor Dislike	Like Slightly	Like Moderately	Like Very Much	Like Extremely

THE SINGLE MOST IMPORTANT

BC111

Please select 1 attribute from the list below that most influenced your rating.

- Too soft
- Firmnes just right
- Too hard
- Too sweet
- Sweetness just right
- Not sweet at all
- Too tart
- Tart just right
- Not tart at all

Next

In this part of the test, you will taste the fruit and be asked to grade the degree of like or dislike and the instensity of some attributes.

Please bite a cracker and drink a sip of water in between samples.

ACCEPTABILITY TEST: OVERALL

SAMPLE BC111

Please LOOK at the blueberries in the petri dishes and express the overall degree of like or dislike of them.
DO NOT EAT the fruit. Take your time to think.

Dislike extremely	Dislike Very Much	Dislike Moderately	Dislike Slightly	Neither Like nor Dislike	Like Slightly	Like Moderately	Like Very Much	Like Extremely

ACCEPTABILITY TEST: COLOR

SAMPLE BC111

Now, focus only in the **color** of the sample and express how much you like or dislike them.

Dislike extremely	Dislike Very Much	Dislike Moderately	Dislike Slightly	Neither Like nor Dislike	Like Slightly	Like Moderately	Like Very Much	Like Extremely

ACCEPTABILITY TEST: FLAVOR

SAMPLE BC111

Focus **only on the FLAVOR** of each sample.
Please **eat all the blueberries that are in the paper cups** at once and express how much you like or dislike them.

Dislike extremely	Dislike Very Much	Dislike Moderately	Dislike Slightly	Neither Like nor Dislike	Like Slightly	Like Moderately	Like Very Much	Like Extremely

THE SINGLE MOST IMPORTANT

BC111

Please select 1 attribute from the list below that most influenced your rating.

- Appearance is appealing
- Appearence is okay
- Appearance is not attractive
- Too soft
- Firmnes just right
- Too hard
- Too sweet
- Sweetness just right
- Not sweet at all
- Too tart
- Tart just right
- Not tart at all

Now, would you order these samples according to you purchasing intention?

(Please drag and drop each sample into the numberd boxes)

Once you are finished, please **sign out.**

This is the end of the test-

1st	2nd	3rd

166 118 330

Next
